# Pima County COVID-19 vaccine solutions dashboard project: lessons learned

**DOI:** 10.3389/fdgth.2024.1345451

**Published:** 2024-04-02

**Authors:** Tina Damalas, Eamon Penney, Theresa Cullen, Aaron Dibner-Dunlap, Cecelia English, Jacob Gomez, Amanda Sapp, Sara Selig, Staci Sutermaster

**Affiliations:** ^1^ Partners in Health United States, Boston, MA, United States; ^2^ Pima County Health Department, Tucson, AZ, United States; ^3^ Surgo Ventures, Washington, DC, United States

**Keywords:** dashboard, data, pima, public health department, vaccine, community vulnerability

## Abstract

Recent improvements in the accessibility of mapping tools and an increased recognition of the importance of leveraging data to inform public health operations has led to enthusiasm among public health departments to rapidly evolve their ability to analyze and apply data to programs. As the COVID-19 pandemic made evident, many health department data systems have been neglected for decades and data literacy among staff low. Significant federal dollars have been allocated to local health departments to modernize health systems. This case study recounts the effort to equip the Pima County Health Department with a highly sophisticated “COVID-19 Vaccines Solutions Dashboard” in 2021–2022, quantifying community vulnerability in the midst of the COVID-19 pandemic and shares key successes and challenges in process and outcomes that can guide other such dashboard initiatives. The experience informed the development of Pima' County Health Department's Data & Informatics Team as well as efforts to cultivate a more robust data culture throughout the department. Many health departments around the United States are in a similar position, and these lessons learned are widely applicable.

## 1 Introduction

When the first widely available COVID-19 vaccinations in the United States were released to the public in early 2021, there were major disparities in vaccine uptake. Longstanding structural inequities that had already led to a disproportionate burden of COVID-19 in certain communities now also caused barriers to vaccine access and uptake. Because of these underlying factors, vaccine eligibility alone did not guarantee equal accessibility. Pima County, Arizona—the state's second-most populous county and one that includes major urban areas such as Tucson, as well as parts of several Native American Nations and Reservations—was no exception to this pattern of pandemic and public health inequity.

Early on in the pandemic, the lack of relevant and transparent data limited the ability of public health actors to determine appropriate local actions effective at averting COVID-19-related morbidity and mortality. Data dashboards that provided various types and layers of data on the many drivers of COVID-19 became a mainstay (1). Their popularity was at least in part accelerated by a well-known dashboard developed by Johns Hopkins University (2). These initial efforts largely focused on showing the extent of the outbreak and public communication (3) but were not geared for guiding targeted intervention based upon hyperlocal context. Dashboards were developed by media outlets or academic institutions rather than local public health authorities (4). As pandemic mitigation methods became understood, local health authorities sought more targeted, specific, actionable tools to inform their efforts. An assessment of 158 dashboards across 53 countries found that the majority did not incorporate social or economic data, only stratified by age and sex, and of those studied, only 12.7% were deemed “highly actionable” (5). In addition, many of these tools were at the zip code or county-level which was not geographically specific enough to meaningfully inform interventions to reach populations most in need.

The Pima County Health Department (PCHD) was one such local health authority that found itself in need of more actionable data visualization tools in order to effectively respond to the health needs of its geographically massive and diverse population of 1.1 million. A consortium of three anchor partners (Pima County Health Department, Partners In Health United States, and Surgo Ventures) sought to create an equity-centered data dashboard, known as the “Vaccine Solutions Dashboard.” This paper will explore that process and the learnings from the dashboard's design and implementation.

## 2 Context

The state of Arizona suffered from some of the world's highest transmission rates throughout the COVID-19 pandemic. By the week ending August 15, 2021, new cases were diagnosed at 278 cases per 100,000 residents per week, increasing at around 24 cases per 100,000 per week; this marked the 10th week of increasing rates (6). Students were preparing to return to school just as a new and dangerous Delta variant-fueled surge was beginning, and measures to protect both students and the general public were heavily politicized. The Governor signed an executive order forbidding cities and counties from imposing vaccine mandates, while the legislature banned schools from imposing mask mandates (7).

This political environment limited the effectiveness of proven prevention and treatment measures and made additional measures unlikely, further endangering the state's most in-need populations (already at risk of greater exposure to COVID-19 and worse outcomes) in particular. With limited policy-level tools available, PCHD needed to creatively bring resources such as testing and vaccines to these communities. PCHD drew upon experiences with program design from their Mitigating COVID in Communities of Color (MC3) program developed in conjunction with the International Rescue Committee, using the Social Vulnerability Index (SVI)^1^. PCHD had previously used SVI to identify parts of the community most at risk; in this situation, SVI was used to identify communities that might benefit from outreach, increase partnership with community organizations, provide education and testing, and coordinate wraparound resources and vaccination efforts. While the MC3 program was ultimately effective, PCHD realized the limitations of the SVI. Indeed, Dr. Theresa Cullen, Pima County Heath Director stated, “SVI was not made for the work we made it do. It was designed by FEMA [for natural disaster response]. Everyone else uses it, so it is a way between health departments to have a common taxonomy and language, but it is not designed to assess communities’ vulnerability with relationship to COVID-19.” This realization emphasized the need for more precise data tools and analytical capacity beyond the typical pandemic-tracking dashboards, and more advanced than basic SVI maps. Precise tools could ensure outreach efforts are specific to community needs and help PCHD to identify communities that SVI alone may have overlooked.

Since June of 2020, a team from Partners In Health United States (PIH-US) has worked with PCHD in southern Arizona to inform equitable public health programming. This work—collaboratively standing up contact tracing and case investigation programs, setting up social support services, and providing data-driven strategic and operational guidance in response to the COVID-19 pandemic—included both short-term data-driven efforts to reach the county's most vulnerable people, and long-term commitments to meet the department's goal of improving its dedication to equity and capacity to manage and leverage data for surveillance, response, analysis, and resilience. This alignment of goals and long-term commitment to health systems strengthening made PCHD and PIH-US natural partners to further leverage data to drive decision making through this new dashboard initiative.

In June 2021, PIH-US was awarded funding through the Health Resources and Services Administration (HRSA) opportunity: Community-Based Workforce to Increase COVID-19 Vaccinations in Underserved Communities, as part of the American Rescue Plan Act of 2021. This grant supported a diverse range of vaccine outreach initiatives for 58 community-organizations in 14 different cities, states, or tribal nations with high social vulnerabilities across the United States. As a part of this portfolio, PIH-US advocated for a portion of the funding to support the general data/analytic capacity needs of PCHD and enabled the department to provide centralized analytical support to the concomitant community-based vaccine outreach efforts occurring under the same funding.

## 3 Program overview and model

As a part of the vaccine equity grant from HRSA, PCHD, Surgo Ventures, and PIH-US began the construction of a “COVID-19 Vaccine Solutions Dashboard”^2^ in July 2021. Surgo Ventures developed specific vaccine tools and technical know-how related to compiling disparate datasets into visually compelling dashboards. PCHD's explicit interest in bolstering internal capacity to leverage data presented an opportunity to test a partnership model wherein PIH-US helped to coordinate the development and execution of a mutually beneficial, timely, and relevant project between two trusted partners. PIH-US acted as the linking strategic and thought partner and served as primary project coordinator, ensuring equity was constantly at the project's center.

The dashboard project had two overarching objectives. The first was to provide a data tool that directly and precisely informed effective, equitable COVID-19 vaccination efforts in Pima County. The second was to serve as a catalyst in the longer-term effort to develop and evolve PCHD's overall capacity to use data to inform and strengthen all facets of its work, from direct intervention design to informing policy and advocacy, and to better measure impact.

The dashboard was intended as a vehicle to enable end users (PCHD staff and leadership) as well as other decision-makers, including PCHD and community-based organizations, to identify vulnerable areas with rising COVID-19 case rates and low vaccination coverage; understand the factors making these communities more vulnerable to the negative health, social, and economic impacts of COVID-19; and understand the possible drivers of low vaccination rates, from poor infrastructure and access to irregular care-seeking behaviors. Ultimately, designers hoped that health department teams and local organizations would use this tool to advocate for appropriately allocated resources and design coordinated, tailored interventions to drive demand for COVID-19 vaccines or improve vaccine access. The dashboard identifies areas that are underperforming or exceeding expectations so the lessons apply to wider efforts to reduce vaccine inequity. As such, the dashboard was designed to support effective and hyperlocal vaccine programming decisions at the census tract level, facilitating precision public health responses. Census tracts are geographical units constructed to include smaller populations of approximately 4,000 people of fairly homogenous population characteristics, economic position, and living conditions (8). Compared to zip code, their use as the primary geographic unit in data collection and analysis offers the opportunity to assess data at a level of disaggregation that can provide a more nuanced understanding of the variability of the pandemic's impact on the county's diverse communities and empowers more targeted decision-making to reach those in highest need.

**Figure 1:**
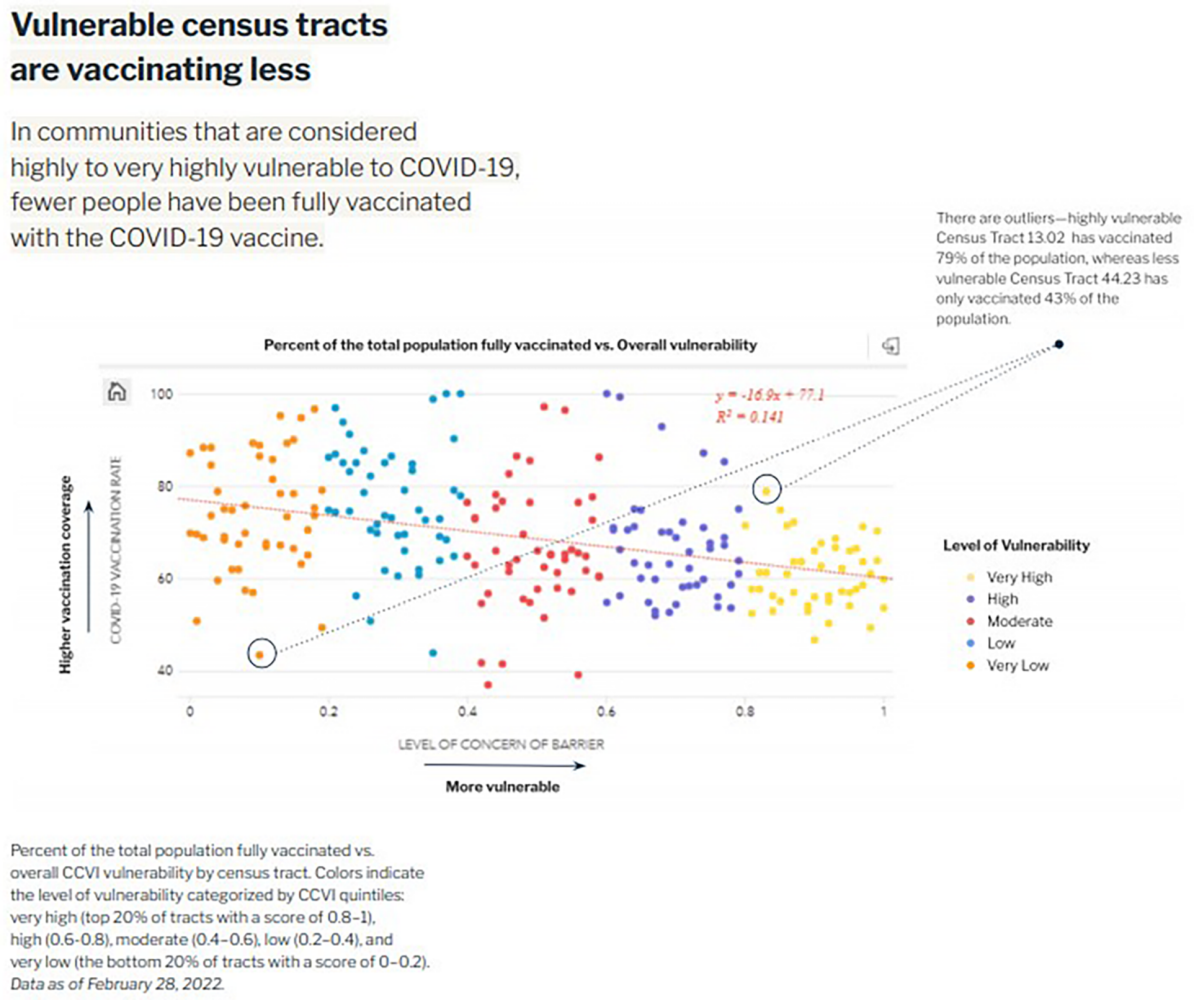
Tracking equity in COVID-19 vaccine coverage.

**Figure 2:**
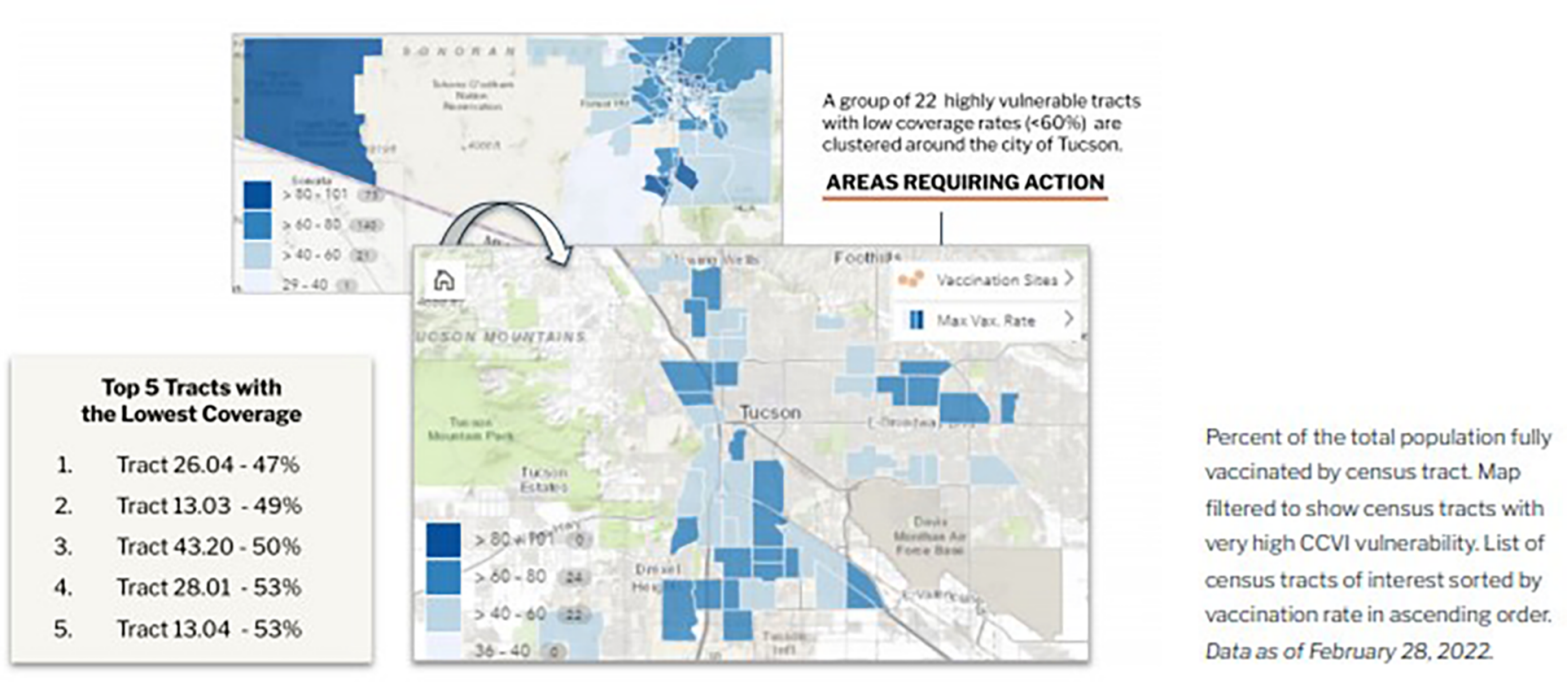
COVID-19 vaccine rate, community vulnerability, and hotspot risk.

The dashboard incorporates relevant data including COVID-19 case rates, vaccination rates, and two indices created by Surgo. The COVID-19 Community Vulnerability Index (CCVI)^3^ captures how and why a community is vulnerable to the health, economic, and social impacts of COVID-19, breaking down the many facets of vulnerability into seven thematic areas: socioeconomic status, minority status and language, household and transportation, epidemiological factors, healthcare system factors, high-risk environments, and population density. The COVID-19 Vaccine Coverage Index (CVAC)^4^ helps contextualize challenges and guides vaccine rollout solutions across census tracts, grouping supply- and demand-related vaccine uptake challenges into five thematic areas: historic undervaccination, social-demographic barriers, resource-constrained healthcare system, healthcare accessibility barriers, and irregular care-seeking behavior. Both indices are designed to not only give a high-level metric of vulnerability by combining relevant indicators into a single measure, but also provide granular insight into the specific characteristics of a given community that drive its vulnerability relative to the pandemic. Dashboard features include the ability to filter census tract maps by different equity criteria based on community vulnerability to pandemic outcomes and hotspot risks (Figure 1). In Figure 1, the dashboard graphs the percent of the population fully vaccinated against overall CCVI vulnerability by census tracts. The resultant visual shows that in the most vulnerable census tracts, COVID-19 vaccination rates were lower than rates in less vulnerable census tracts, clearly correlating equity levels with vaccine coverage. These insights allow public health decision-makers to design outreach initiatives in collaboration with community partners to address the specific needs of a given community (Figure 2). Figure 2 exemplifies one way in which the dashboard may be used to identify census tracts requiring attention by the health department. In this example, the tool shows the percent of the total population that is fully vaccinated by census tract; the map is further filtered to show census tracts with especially high CCVI vulnerability. The resultant overlay produces a list of top census tracts with both high vulnerability and low vaccine coverage, creating a priority list for health department intervention.

The dashboard development process took place between December 2021 and April 2022. During this process, various versions of the dashboard were presented to PCHD and Pima County leadership, PCHD project teams, and community-based organizations to socialize the tool and to gain feedback for continuous improvement. In addition, Surgo presented the dashboard to both the PCHD Community Advisory Committee and the Pima County Ethics Committee, engaging in a dialogue around the value of its public availability and identification of potential unintended consequences.^5^

In April 2022 the design and development phase of the dashboard concluded. The process began of transferring the full ownership of the dashboard from Surgo Ventures to the Pima County GIS team. The dashboard currently is live and publicly available.^6^ 

## 4 Results & discussion

The project had mixed results in achieving its objectives. With regards to the first—directly informing PCHD's outreach and efforts in the short term—a number of challenges discussed in more detail in the first section below limited success. However, the process of designing, developing, and implementing the dashboard was incredibly instructive for PCHD teams and leadership. The broader ways in which this project accelerated the evolution of PCHD's use of data are described in the second section below.

As the dashboard was developed in the context of a major public health emergency and subject to the timing and staffing pressures associated with that crisis, a robust evaluation was not plausible. However, feedback and validation from users across a diverse array of teams was gathered via key informant interviews with members of PCHD, the project teams, and other organizations as noted in the following sections.

### 4.1 Objective 1: provide a tool to directly inform the operationalization of effective and equitable COVID-19 outreach and vaccination efforts

As a tool, the dashboard functioned as designed in that it effectively used Surgo to both identify and provide insights into Pima County's most at-risk communities and overlaid that information with up-to-date statistics on COVID-19 infection and vaccination rates. However, a number of process gaps (both within and outside of the project team's control) prevented the dashboard from being a meaningful tool to inform PCHD team's day-to-day operations.

#### 4.1.1 End user bandwidth—lack of adherence to best-principles for user-centric design

End user and project team bandwidth was the main barrier to the dashboard informing public health programming in real time. In particular, the core design and rollout phase of the dashboard coincided with especially high-demand times for PCHD staff: concurrent waves of high COVID-19 transmission, critical phases of vaccine rollout, resumption of in-person activities in the community, and significant politicization of public health measures. Therefore, PCHD staff, the end users, were often unable to engage thoroughly in an iterative development process. The project team was also stretched onto numerous projects, limiting the ability to adhere to best practices in digital design.

This lack of engagement led to some predictable issues. The design of the dashboard was not as intuitive or user-friendly as teams needed, nor was it specific enough to the work of any given team. Already-stretched teams lacked bandwidth to steward its adoption, meaning they were often unable to incorporate the dashboard's more nuanced features into their work.

#### 4.1.2 Lack of flexibility—the changing nature of the pandemic and pandemic response made certain dashboard variables irrelevant and limited the ultimate effectiveness of the tool

The rapidly evolving COVID-19 landscape limited the effectiveness of the dashboard, as shifting dynamics of the pandemic necessitated changes in the dashboard to ensure its relevancy. While the CCVI and CAVC effectively visualized vulnerability and the drivers of vulnerability and this remained static, other outbreak-specific dashboard elements did not. Perhaps the most important challenge was the evolution from universally reported PCR testing to at-home rapid testing. With PCR testing the results of the vast majority of tests were reported to the state, providing strong visibility into case occurrence and location. The shift to at-home testing made COVID-19 case rate data much less valuable. Additionally, definitions used to create categorical standards changed. For example, during the design stage, the dashboard's hotspot feature (which allows the user to visualize and incorporate disease rates and see where the pandemic was hitting hardest) became less relevant as case rates dropped and the standard for “hot” shifted/The dashboard was not easily adaptable.

Definitions and terms for vaccine eligibility also evolved over time. The definition of “fully vaccinated” continued to change, as did age-group eligibility. As boosters became available, the CDC definition of “fully vaccinated” shifted, as did the ages and populations who were vaccine-eligible. The dashboard operated around the original terms and eligibility; it was not able to account for the evolution, making the dashboard less useful by the time it launched. Future dashboard projects should consider predicting possible changes to a tool's scope and use and enable maximum flexibility in the design process.

#### 4.1.3 Cultural mismatch—the dashboard design did not directly meet needs of the end user across teams, and lacked an adequate understanding of PCHD team data culture or expectations

Some PCHD teams were able to use the dashboard as intended, but not to the extent hoped, as each had a distinct set of needs and expectations specific to their own work streams. Several teams used it to identify vulnerable geographic areas but did not utilize the features that enable users to investigate or identify the drivers behind that vulnerability to inform effective vaccine programming or outreach efforts. Some resorted to using the dashboard for initial information, and then supplementing this by laying over other mapping tools that included available resources or the location of community-based organizations. As such, many teams viewed the dashboard as too unwieldy to incorporate into their work. This was in part because of an overload of available data within the dashboard. “We didn’t know which layers were most significant for a given geography, and that's a huge issue,” said Dr. Cullen. “When there are 20 different things the user can query, and they don’t know which one is most relevant, they end up not doing anything.”

Along with data overload, there were also misaligned expectations regarding the scope of the dashboard and what data sets were available. Teams whose work primarily entailed organizing vaccination and outreach events cited the need to be able to pull relatively nuanced information (such as local community actors with contact information or historical data on past vaccination outreach efforts). Others wished the dashboard allowed them to download, manipulate, and independently analyze data sets—functions that go far beyond the dashboard's scope.

Designers should have taken into account these expectations, as well as team capacity and the full array of expected uses at the start of the dashboard project, with more consideration for human-centered design. PCHD partners should have been more involved in the process to ensure the dashboard's end product was not only applicable, responsive, and useful, but also that it could be adapted and maintained in accordance with PCHD's constantly evolving needs. During the initial scope of the project, designers believed that after the Surgo team's initial buildout, PCHD would be able to assume ownership and customization responsibilities of the dashboard. This proved to be unrealistic. The gap between PCHD's actual data capacity and the level needed to own and modify the dashboard proved larger than originally foreseen and is discussed later in this paper.

### 4.2 Objective 2: strengthen PCHD's use, understanding, and ability to leverage data analysis and tools

Despite the challenges developing a tool that directly informed project teams' day-to-day decision-making, the dashboard project did succeed in unexpected ways at meeting the second objective: strengthening PCHD's use, understanding, and ability to leverage data in its work. Learnings and outcomes from the experience and the evolution of its operationalization advanced data use within and across PCHD. The dashboard project fast-tracked collaboration and equipped teams with greater data literacy and the ability to apply data to projects.

#### 4.2.1 Interaction with the dashboard promoted connections and communication within and across previously unconnected PCHD teams, fostering an environment of cooperation and the potential for applying data analytics

The dashboard project helped the brand-new Data & Informatics team build relationships internally at PCHD as well as with other Pima County offices. Within PCHD, one project lead said, “The dashboard really opened up a lot of relationship building and explored a path forward that wasn’t there before.” For example, discussions stemming from the dashboard led the PCHD Tobacco program team to inquire about leveraging data to target focus areas for a program identifying vulnerable areas in proximity to early childhood education centers. These explorations to creatively use data led to questions about data sharing and creating a centralized data hub that would be accessible to employees across all teams. This could launch further opportunities for learning, adaptation, efficiency, and evidence building both at the individual team and organizational levels.

“The Surgo project was the beginning of the relationship between the PCHD data team and the GIS team. We didn’t ever have interaction before this.” —Amanda Sapp, PCHD Data & Informatics Program Manager

As a single department within the broader county government structure, PCHD's ability to achieve its broader goals with regard to data is not entirely within its own hands. Like many county public health departments, other departments own various data sets, the IT department hosts the GIS team, and before this project some dashboarding had been done but it was generally single-layer and not always public. This project increased communication and transparency across teams and has encouraged data sharing and transferable solutions in the form of collaborative programming and learning.

“*When we started this process, we were fairly unsophisticated about data…this project let us see the potential for dashboarding.*”—Dr. Theresa Cullen, Pima County Health Director

#### 4.2.2 PCHD staff are now better equipped to effectively advocate for impactful partnerships, investment in health technology, and data integration in PCHD work

Spurred by the experience of dashboard design and operationalization, PCHD staff cited an increased ability to advocate for themselves, their teams, and the importance of data in the future. While the dashboard may have fallen short of some specific expectations due in part to capacity constraints described previously, PCHD teams feel they have identified many of the underlying issues contributing to this outcome and are able to ask appropriate questions when they encounter similar opportunities in the future. As such, this awareness will improve their ability to effectively establish project plans with partners.

Experience from this project has also helped team leaders advocate for funding for data and technology. Leaders have advocated internally for increased data-oriented hiring on several teams, growing departmental capacity and data literacy. They have also become more competitive applicants for grants and outside funding opportunities as a result of the knowledge gained throughout this project. One team lead said, “I can now chase a grant and feel equipped to make my case. Going through this process shed a lot of light on how I can speak to that.”

#### 4.2.3 Data from the dashboard improved training opportunities for staff and partners, especially with regards to understanding and identifying vulnerability

As described, this project catalyzed increased collaboration between teams. However, it also revealed a need for increased training across the department. Program teams that understood how to phrase questions in ways that could be answerable by data analyses were able to launch new partnerships, while other teams first needed to build a common vocabulary and understanding around data. The inverse of this dynamic also applied: the data team needed better understanding of program teams' work so that they could develop and support with tools that were most impactful.

A second way the dashboard catalyzed training was through the dashboard itself. Its quantification of vulnerability through the CCVI and CVAC provided opportunities for internal and external training with PCHD staff and partners as to how to quantify vulnerability and draw connections between those metrics and what is driving them. For example, the dashboard was used for the community health worker vaccine access and training program to train workers not only on vulnerability and what that looks like in Pima County, but also to strategize around how and where to best target programming aimed at increasing uptake of booster shots.

### 4.3 Lessons learned

In addition to more targeted learnings related to the goals outlined above, this project brought to light several key overarching lessons. These insights may be useful to apply not only to future PCHD data-oriented endeavors, but also to data-focused projects and partnerships more broadly.

#### 4.3.1 Data is a starting point for equity, but must be paired with community engagement to result in effective programming

The COVID-19 crisis drew much-needed attention to some of the many longstanding structural inequities that have created and reinforced classes of people and communities and divergent health and wellness outcomes in the U.S. This created an opportunity for data to inform and direct interventions and programming—to be a part of the solution. Teams scrambled to create dashboards and data indices to ensure the necessary aggregated data was collected and correctly applied. While this development prioritizing data in an effort to bring about equity was a welcome change, quantitative data alone cannot solve this problem.

Instead, decision-makers and program designers should apply high-quality data in conjunction with thoughtful community engagement. Data must be married with community input and used to complement substantive community engagement—not as a replacement. Communities and those who work closely within communities can provide context and perspective that data simply cannot reveal. Even the most perfect dashboard that can steer interventions in the right direction lacks the full narrative that community knowledge can offer. Therefore, health departments and other leading actors should depend heavily on feedback garnered from deep and trusting relationships with communities to define and prioritize goals and objectives for any data-informed programming.

#### 4.3.2 Dashboards should consider stakeholders and be based on an understanding of how data points correlate to health outcomes—as well as how various stakeholders can contribute

In the wake of the COVID-19 pandemic, dashboards have become popular and accessible to health departments and organizations around the country. To execute dashboards well and use them to their maximum impact, health departments should carefully consider the stakeholders (internal and external) involved when constructing the dashboard and framing communication around their purpose and use. With this in mind, health departments should also evolve their data sophistication, broadly advancing from clunky and less effective SVI to more relevant indices (e.g., CCVI) and fewer, more targeted data points. They should also establish a better understanding of how variables correlate to health outcomes specifically; external stakeholders may help hone data points to avoid data overload.

“Everyone thinks 100 data points are better than five, but they overload people and limit use of the tool itself.”—Dr. Theresa Cullen, Pima County Health Director

#### 4.3.3 Impactful integration of data solutions requires both dedicated teams or individuals to facilitate adoption, as well as policies in place that ensure both adoption and adaptation

Improving any health department's ability to leverage data to drive programs and policy effectively is not as simple as just providing tools or hiring data scientists. Successful application of data to public health requires deliberate policy, as well as both the data scientists and analysts to sustain, and ongoing and dynamic collaboration with project teams and public health leaders to evolve. This should be a bi-directional conversation: data scientists must understand the needs of the program managers, while program managers must understand foundations of data science to engage and pragmatically understand how best to apply data insights to their work. Just as they should facilitate person-to-person collaboration, health departments must also establish and follow policies around data use. A tool or dashboard cannot simply be handed over to teams with the expectation that it will be used consistently and have impact. Policies regarding their use, training, review, and adaptation need to be established collaboratively and communicated clearly. Interdisciplinary or inter-departmental working groups or “task forces” can oversee this process and ensure open lines of communication are systematized.

## 5 Conclusion & constraints

While this paper and the associated evaluation of the dashboard's implementation and success does have constraints (it is qualitative in nature, informed largely by observation and key informant interviews rather than by quantitative methods, and is evaluated largely by actors who were involved in the design and implementation of the dashboard), the development and implementation process of the “Vaccine Solutions Dashboard” in Pima County provided meaningful learning opportunities for both PCHD and partners including PIH-US and Surgo Ventures, and beyond. In the future, it may be possible to scale this dashboard and others like it to identify inequities in other public health areas of interest. Specifically, health departments and their partners may be able to substitute different outcomes or add possible explanatory factors to the dashboard to broaden the scope of programmatic solution design for richer and more far-reaching potential in Pima County and beyond.

The experience demonstrated that (1) PCHD employees recognized the tremendous need to integrate data into their work to effectively create programming for the communities they serve—and enthusiastically embraced its potential to change the landscape, and (2) the experience of dashboard development and implementation allowed PCHD leadership to break down the lofty goal of becoming a more data-informed health department into actionable steps. This notion can often be somewhat nebulous, but this process offered an opportunity to identify the concrete actions that contribute to this goal. This included increasing data literacy among staff, increasing in-house technical expertise, developing forums for sharing and collaboration, and other areas. PCHD is now more strongly positioned to serve its community members today and into the future and achieve its mission: To promote, protect, and preserve health in Pima County through leadership, services, education, and partnerships.

## Data Availability

The original contributions presented in the study are included in the article/Supplementary Material, further inquiries can be directed to the corresponding author.

## Footnotes

^1^ https://www.atsdr.cdc.gov/placeandhealth/svi/index.html

^2^ https://surgoventures.org/resource-library/covid-19-vaccine-solutions-dashboard-leveraging-granular-data-to-improve-covid-19-vaccination-coverage-in-pima-county-arizona

^3^ https://surgoventures.org/resource-library/report-vulnerable-communities-and-covid-19

^4^ https://vaccine.precisionforcovid.org/

^5^Ultimately, the committees decided that the risk of harm to the Pima community was quite low, but the dashboard incorporated their recommendations to add contact information for the PCHD data team so that community members could contact team members if necessary. It was also agreed that while this tool applied to the context of COVID-19 had relatively low risk, if the dashboard was later adapted to focus on other health outcomes, PCHD and partners would re-evaluate risk to protect against promoting stigmas.

^6^ https://insights.arcgis.com/#/view/350cf115ee9a445d87171dd779108edb
